# Advances in artificial intelligence-driven MRI research in Alzheimer’s disease

**DOI:** 10.3389/fneur.2026.1943265

**Published:** 2026-10-08

**Authors:** Lihua Deng, Yan Zeng, Peiling Ou, Suyi Zhou, Yu Gou, Cong Huang

**Affiliations:** 1 Department of Radiology, The First People’s Hospital of Neijiang, Neijaing, Sichuan, China; 2 Department of Radiology, No. 926 Hospital, Joint Logistics Support Force of PLA, Kaiyuan, Yunnan, China; 3 7T Magnetic Resonance Imaging Translational Medical Center, Department of Radiology, Southwest Hospital, Army Medical University (Third Military Medical University), Chongqing, China; 4 Department of Ultrasound, The First People’s Hospital of Neijiang, Neijiang, Sichuan, China; 5 Department of Radiology, Graduate School of Peking Union Medical College, Chinese Academy of Medical Sciences and Peking Union Medical College, Beijing, China; 6 Department of Nuclear Medicine, The First People’s Hospital of Neijiang, Neijiang, Sichuan, China

**Keywords:** Alzheimer’s disease, artificial intelligence, deep learning, early diagnosis, image analysis, machine learning, magnetic resonance imaging

## Abstract

Alzheimer’s disease (AD), the most prevalent neurodegenerative disorder, poses substantial challenges for early diagnosis and longitudinal monitoring—two cornerstones of effective clinical management and therapeutic intervention. Magnetic resonance imaging (MRI), owing to its non-invasiveness and high spatial resolution, has emerged as an indispensable tool in AD research. In recent years, artificial intelligence (AI), particularly deep learning (DL) and machine learning (ML) techniques, has demonstrated remarkable potential for automating MRI analysis, extracting subtle imaging features, and supporting objective disease prediction. This review comprehensively summarizes the latest advances in AI-driven MRI applications for AD diagnosis, staging, prognosis, and adjuvant therapeutic monitoring. It highlights key progress in AI model development, multimodal data processing, and clinical translational value. Furthermore, current bottlenecks, including data heterogeneity, model interpretability, and real-world deployment, are systematically discussed, and future directions are outlined to enhance the precision and clinical utility of AI-assisted MRI in AD. Overall, AI-MRI has evolved from laboratory validation toward early clinical adoption, with explainability and multi-center generalizability identified as critical priorities in the next research phase.

## 1 Introduction

Alzheimer’s disease (AD) is a progressive neurodegenerative disorder predominantly affecting the elderly population, characterized by a gradual decline in cognitive functions such as memory, language, and executive abilities. This deterioration severely impairs patients’ quality of life and imposes substantial burdens on caregivers and healthcare systems. As the most common form of dementia in old age, AD lacks curative treatments, making early diagnosis and timely intervention critical for slowing disease progression—an unmet clinical need that AI-MRI aims to address.

Traditionally, the diagnosis of AD has relied heavily on clinical evaluation and neuropsychological testing, which, while informative, are often subjective and may detect the disease only after significant neurodegeneration has occurred. Early and accurate diagnosis remains a critical challenge, as it is essential for timely intervention and management strategies aimed at slowing disease progression. Magnetic resonance imaging, a non-invasive imaging modality, has emerged as a pivotal tool in the early detection and monitoring of AD due to its ability to provide detailed structural and functional information about the brain. MRI can reveal characteristic patterns of brain atrophy, particularly in regions such as the hippocampus and entorhinal cortex, which are among the earliest affected areas in AD pathology. Moreover, advanced MRI techniques, including functional MRI (fMRI), diffusion tensor imaging (DTI), and magnetic resonance spectroscopy (MRS), offer insights into functional connectivity, white matter integrity, and metabolic changes, respectively, further enriching the understanding of AD-related brain alterations.

Despite these advances, the interpretation of MRI data for AD diagnosis and prognosis is complex, requiring expert knowledge and often involving subjective assessments. Crucially, AD exhibits overlapping imaging features with other dementias (e.g., frontotemporal dementia, Lewy body dementia), and early pathological changes are subtle and easily overlooked by human observers, exacerbating diagnostic ambiguity and inter-rater variability. In recent years, artificial intelligence, particularly deep learning (DL) methodologies, has revolutionized medical image analysis by enabling automated, objective, and high-throughput processing of complex imaging data. AI-driven approaches can extract subtle imaging features that may be imperceptible to human observers, thereby enhancing diagnostic accuracy and enabling early detection of AD (1, 2). Deep convolutional neural networks (CNNs), for instance, have demonstrated remarkable performance in classifying MRI scans into AD, mild cognitive impairment (MCI), and cognitively normal categories, facilitating the identification of disease stages and progression trajectories (3, 4). Furthermore, AI models have been developed to predict cognitive decline and disease progression by integrating MRI data with clinical and biomarker information, supporting personalized medicine approaches (5, 6).

The integration of AI with MRI not only improves diagnostic precision but also aids in the extraction of imaging biomarkers that reflect underlying neuropathological changes. For example, AI techniques have been applied to quantify brain atrophy patterns, white matter hyperintensities, and functional connectivity alterations associated with AD. Additionally, multimodal imaging approaches combining MRI with positron emission tomography (PET) and other modalities have been enhanced by AI algorithms to provide comprehensive assessments of amyloid deposition, neurodegeneration, and vascular contributions to cognitive impairment. These advancements hold promise for improving early diagnosis, monitoring therapeutic responses, and stratifying patients in clinical trials.

However, challenges remain in the widespread clinical adoption of AI-driven MRI analysis for AD. These include the need for large, diverse, and well-annotated datasets to train robust models, the interpretability of AI decisions, and the standardization of imaging protocols and analysis pipelines. Moreover, while deep learning models excel in feature extraction, some studies suggest that traditional approaches focusing on predefined brain regions may perform comparably in predicting disease progression (7, 8). Future research directions emphasize the development of explainable AI models, multimodal data integration, and longitudinal studies to capture the dynamic nature of AD pathology.

Against a growing body of recent review articles covering AI-MRI for Alzheimer’s disease published from 2020 to 2026, the present narrative review has several distinguishing features. First, this review balances algorithm-oriented technical summary, imaging-biomarker biological interpretation and real-world translational bottlenecks, instead of only reporting model performance metrics. Second, we emphasize dataset demographic bias, especially the under-representation of Asian cohorts within mainstream public AD neuroimaging datasets, an issue insufficiently highlighted in most existing reviews. Third, we provide an evidence-based phased translational roadmap spanning 2026–2030 for AI-MRI in AD clinical practice. Fourth, systematic comparative analysis among traditional machine-learning, CNN, Transformer and GNN approaches, as well as multimodal fusion strategies, are integrated, with explicit discussion of the large performance gap observed between laboratory-trained models and real-world clinical requirements. This review focuses on AI-driven MRI analysis for AD, with emphasis on early diagnosis, risk stratification, progression monitoring and multimodal integration.

In summary, the convergence of artificial intelligence and magnetic resonance imaging represents a transformative advancement in AD research and clinical practice. AI-driven MRI analysis enhances the early detection and accurate diagnosis of AD, facilitates the identification of novel imaging biomarkers, and supports personalized prognostic assessments. This review systematically synthesizes the latest progress of AI-MRI in AD early diagnosis, staging, prognosis, and multimodal integration, dissects core bottlenecks in data quality, model generalization, and clinical translation, and proposes actionable future directions—providing a panoramic reference for advancing precision AD diagnosis. Continued interdisciplinary efforts are essential to overcome current limitations and fully realize the potential of AI-powered neuroimaging in combating this devastating disease.

## 2 Application of artificial intelligence technology in MRI image processing

### 2.1 Application of deep learning models in automatic segmentation of MRI images

Convolutional neural networks and their variants have demonstrated significant advantages in automatic segmentation of brain regions from MRI images. CNNs excel in learning hierarchical features directly from raw image data, enabling precise delineation of complex brain structures without manual intervention. For instance, U-Net and its derivatives leverage an encoder-decoder architecture that captures multi-scale contextual information, which is critical for segmenting brain tissues with varying shapes and sizes. The ability of CNNs to preserve spatial resolution through skip connections enhances edge localization, a key factor in brain region segmentation.

Among commonly used architectures, U-Net and ResNet have been widely adopted for segmenting AD-related brain regions such as the hippocampus and cortical areas. U-Net’s symmetric structure with multi-scale feature extraction facilitates accurate segmentation of gray matter (GM), white matter (WM), and cerebrospinal fluid (CSF), which are crucial for AD diagnosis. Residual networks (ResNet) improve gradient flow and enable deeper architectures, enhancing feature representation for subtle pathological changes. AD-specific segmentation faces unique challenges: blurred hippocampal boundaries, severe ventricular enlargement, uneven gray/white matter atrophy, and low contrast in low-field MRI, which limit the performance of generic segmentation models. Studies have shown that these architectures achieve high Dice similarity coefficients (DSC) in segmenting AD-relevant brain structures, supporting their clinical utility.

Segmentation accuracy directly impacts the quality of subsequent feature extraction and diagnostic modeling. Precise delineation of brain tissues ensures that extracted biomarkers, such as volumetric measurements of the hippocampus or cortical thickness, are reliable and reproducible. Inaccurate segmentation propagates errors downstream, reducing the sensitivity and specificity of AD classification models by distorting core quantitative indicators of neurodegeneration. For example, improved segmentation accuracy has been linked to enhanced classification performance in distinguishing normal, mild cognitive impairment, and AD subjects using CNN-extracted features combined with machine learning classifiers (9). Therefore, advancements in segmentation networks contribute substantially to the overall diagnostic pipeline.

Recent innovations include mixed-scale asymmetric convolutional blocks and multi-branch feature extractors that further refine segmentation performance by capturing isointense tissue boundaries and preserving edge details. Specifically, attention-gated U-Net introduces attention gating modules to suppress irrelevant background voxels and focus computation on target brain regions such as the hippocampus; residual skip connections mitigate gradient degradation when network depth increases. Multi-scale fusion modules aggregate feature maps from different encoder layers to resolve blurred tissue boundaries induced by severe hippocampal atrophy and ventricular dilation in AD patients. Nevertheless, these improved segmentation networks still face practical obstacles: segmentation performance degrades obviously under low-field MRI with low tissue contrast, and domain shift across multi-center datasets remains a non-negligible limitation for clinical deployment. Targeted improvements for AD segmentation, such as attention-gated U-Net, residual skip connections, and multi-scale fusion modules, have addressed boundary ambiguity and improved robustness to atrophy-induced tissue distortion. Additionally, integrated frameworks combining segmentation and tumor lesion detection have been developed, demonstrating adaptability across different MRI contrasts and resolutions.

In summary, deep learning models, especially CNN-based architectures like U-Net and ResNet, have become indispensable tools for automatic segmentation of MRI brain images in AD research. Their ability to accurately segment critical brain regions enhances downstream analyses, including feature extraction and disease classification, thereby improving early diagnosis and monitoring of AD.

### 2.2 The role of machine learning methods in MRI feature extraction and selection

Traditional machine learning algorithms such as Support Vector Machines (SVM) and Random Forests (RF) have been extensively applied in the selection and classification of MRI-derived features for AD diagnosis. SVM, in particular, has demonstrated favorable performance in distinguishing AD from frontotemporal dementia (10). These algorithms rely on handcrafted features, which often require expert knowledge for effective extraction, posing challenges in scalability and automation (11). Nonetheless, their interpretability and relatively lower computational demands make them valuable in clinical settings.

Multidimensional feature construction incorporating texture, morphological, and volumetric characteristics has been pivotal in enhancing diagnostic accuracy. Texture analysis captures subtle signal variations in MRI that are not visually apparent, providing quantitative markers of tissue heterogeneity associated with AD pathology (12). Morphological features, such as cortical thickness and shape metrics, alongside volumetric measures of critical regions like the hippocampus and entorhinal cortex, contribute complementary information reflecting neurodegeneration (13, 14). Combining these diverse features enables a more comprehensive representation of brain changes, improving the sensitivity and specificity of machine learning models.

Feature selection methods play a crucial role in improving model generalization by reducing dimensionality and mitigating overfitting. Common feature-selection strategies applied in AD-MRI radiomics include filter-based, wrapper-based and ensemble-driven approaches. Filter-based methods rank features according to statistical metrics without involving downstream classifiers, featuring fast computation but ignoring feature interaction effects. Wrapper-based methods embed classifier performance into feature subset searching, yet bring heavy computational burden and high overfitting risk on small cohorts. Ensemble-based feature selection fuses outputs from multiple selection algorithms, yielding more stable and robust feature subsets and has gained wide adoption for high-dimensional brain imaging radiomics. Techniques such as ensemble-based feature selection have been employed to identify the most discriminative features from text data (15). For example, a two-stage framework integrating U-Net generated voxel-level hierarchical maps with graph convolutional neural networks has enhanced classification performance (16). Moreover, selecting features that are biologically plausible and consistent with known AD pathology supports the clinical relevance of the models. Effective feature selection thus facilitates robust and generalizable machine learning models applicable across diverse datasets.

Recent advances have also seen the integration of multimodal data, combining MRI features with genetic and clinical information to improve predictive accuracy. Multimodal approaches leverage the complementary strengths of different data types, with MRI capturing anatomical changes and genetics providing risk stratification prior to symptom onset (17). Machine learning models that incorporate such multimodal features have demonstrated superior performance in predicting disease progression and conversion from mild cognitive impairment to AD. This underscores the importance of sophisticated feature extraction and selection strategies in harnessing the full potential of MRI data within machine learning pipelines.

To clarify the positioning of different technical routes, Table 1 systematically compares traditional machine learning, CNN-family deep learning and Transformer-based models from multiple technical dimensions, highlighting their respective mechanism, data requirement and applicable scenarios for AD-MRI analysis:

**Table 1:** Comparison of traditional machine learning, CNN-based deep learning and transformer-based models for AD-MRI analysis.

Dimension	Traditional machine learning (SVM/RF)	Convolutional neural networks (CNN family)	Transformer-based models
Feature extraction	Manual feature engineering, expert-driven	End-to-end automatic local feature extraction	Global self-attention-driven feature learning
Core modelling mechanism	Statistical classification on handcrafted features	Local receptive-field convolution operation	Multi-head self-attention for long-range dependency capture
Interpretability	High; feature traceable	Moderate; requires post-hoc explainable-AI tools	Low-to-moderate; attention-map assisted interpretation
Data requirement	Stable under small annotated sample size	Requires moderate-to-large annotated datasets	Demands large-scale annotated datasets
Long-range dependency modelling	Not applicable	Limited; relies on stacked convolution	Strong; captures global brain spatial relationships
Performance characteristics	Moderate ceiling; constrained by handcrafted feature quality	Excellent for local tissue/lesion pattern mining	Superior for global brain network and volumetric 3D-MRI analysis
Hardware and computational cost	Low	Moderate	High; large GPU memory consumption
Typical AD-MRI application	Radiomics feature classification, risk stratification	Brain tissue segmentation, local atrophy classification	3D volumetric MRI classification, functional connectivity analysis

SVM, support vector machine; RF, random forest; GPU, graphics processing unit.

In summary, traditional machine learning methods remain foundational in MRI feature extraction and selection for AD diagnosis. The construction of multidimensional features encompassing texture, morphology, and volume, combined with rigorous feature selection techniques, enhances model performance and generalizability. Integrating multimodal data further augments predictive capabilities, paving the way for more accurate and early diagnosis of AD.

### 2.3 Construction of semi-automated and automated workflows

The integration of semi-automated workflows that combine expert knowledge with artificial intelligence algorithms offers significant advantages in AD diagnosis using MRI. Semi-automated approaches leverage the interpretability and domain expertise of clinicians while harnessing AI’s capacity for high-throughput data processing and pattern recognition. This synergy enhances diagnostic accuracy by reducing human error and variability, while maintaining clinical oversight to validate AI outputs. For example, convolutional neural networks can automatically extract hierarchical features from raw MRI data, minimizing the need for extensive preprocessing (18).

Recent studies demonstrate that such workflows improve clinical efficiency and reduce misdiagnosis rates. Deep learning models, such as ensembles of U-Nets combined with graph convolutional networks, have been employed to generate voxel-level disease severity maps and classify AD stages with high accuracy. These semi-automated frameworks enable localization of abnormal brain regions and provide interpretable outputs (16). Moreover, the proposed EADCD-TIPAIT technique based on MRI imaging achieved a classification accuracy of 95.00% (19).

The use of multi-center datasets is critical for training robust AI models that generalize well across diverse populations and imaging protocols. Multi-center data introduce heterogeneity in scanner types, acquisition parameters, and patient demographics, which can challenge model performance. Addressing this heterogeneity requires sophisticated preprocessing and domain adaptation techniques. For instance, convolutional adversarial autoencoders have been developed to reduce inter-center variations by aligning MRI scans into a common space, thereby improving classification accuracy across centers (20). Such approaches ensure that AI models are not biased toward specific datasets and maintain reliability in real-world clinical applications.

Handling data heterogeneity also involves leveraging large-scale, multi-modal datasets to capture the complex pathophysiology of AD. Studies employing multimodal MRI data combined with clinical and biomarker information have shown that integrating heterogeneous data sources enhances predictive performance and diagnostic precision. Transfer learning strategies help evaluate approaches to address data limitations, and ensemble learning classification models have obtained excellent performance after validation on multiple datasets. These advances underscore the importance of comprehensive data integration and preprocessing in constructing effective semi-automated and automated workflows for AD diagnosis (Figure 1 illustrates the standard AI-MRI workflow for AD diagnosis).

**Figure 1:**
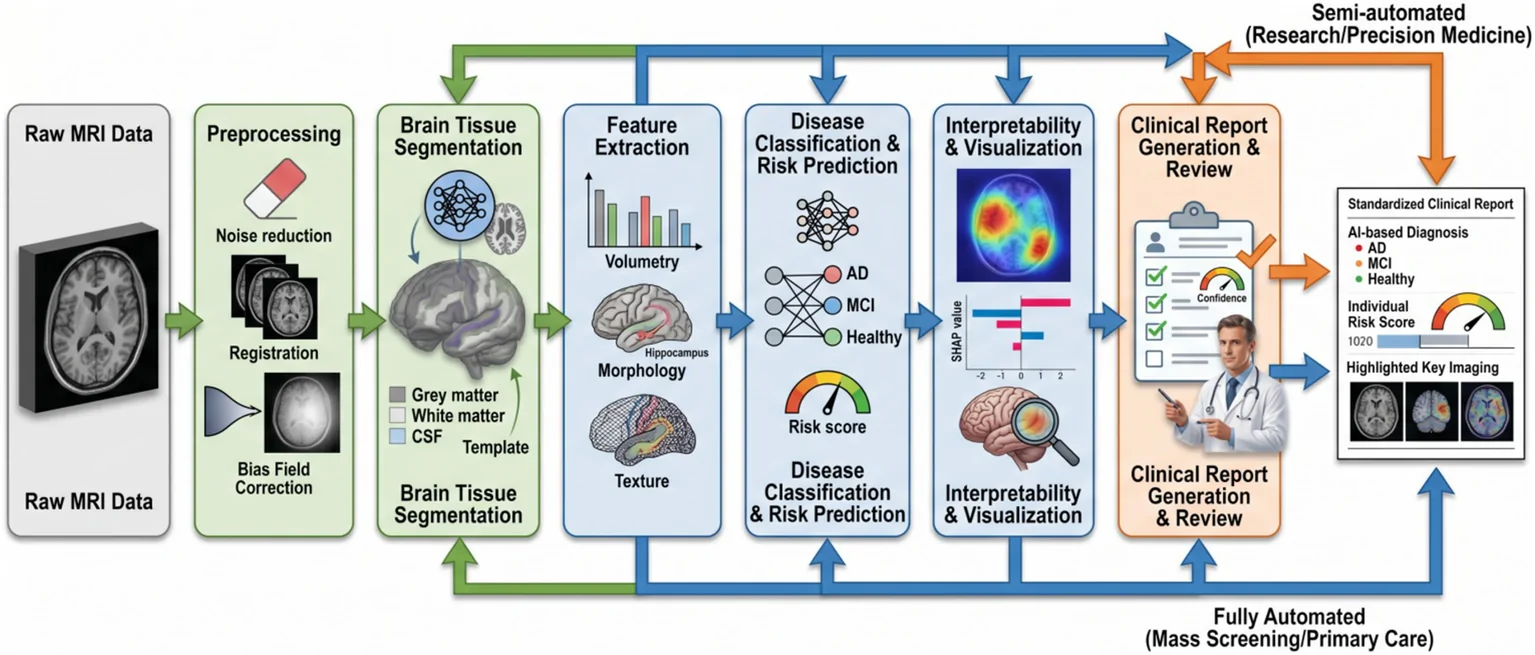
Standard workflow of artificial intelligence (AI)-assisted magnetic resonance imaging (MRI) analysis for Alzheimer’s disease (AD) diagnosis. The pipeline comprises seven sequential stages: raw MRI data acquisition, preprocessing (including noise reduction, registration, and bias field correction), brain tissue segmentation, feature extraction (covering morphological, textural, and volumetric metrics), disease classification and prognosis prediction, visualization and explainability analysis, and final clinical reporting. Two operational modes are presented: semi-automated workflow requiring clinician validation of intermediate outputs, suitable for research and precision diagnosis; and fully automated workflow enabling end-to-end AI processing, optimized for large-scale screening and primary care applications.

In summary, semi-automated workflows that integrate expert knowledge with AI algorithms optimize the balance between automation and clinical interpretability, improving diagnostic efficiency and accuracy. The incorporation of multi-center datasets and strategies to manage data heterogeneity are essential to develop robust, generalizable models suitable for widespread clinical deployment. These developments pave the way for AI-driven MRI analysis to become a reliable tool in the early detection and management of AD.

## 3 Research progress of AI-driven MRI in the early diagnosis of AD

### 3.1 Identification of imaging characteristics in early pathological changes

AD is characterized in its early stages by subtle but critical structural brain changes detectable via magnetic resonance imaging. Among these, hippocampal atrophy and cortical thinning stand out as hallmark imaging features reflecting neurodegeneration. Hippocampal volume reduction is consistently reported as a sensitive biomarker for early AD, correlating with memory impairment and disease progression. Cortical thinning, particularly in temporal and parietal lobes, further signifies neuronal loss and synaptic dysfunction. These structural alterations precede overt clinical symptoms, making their accurate detection essential for timely diagnosis and intervention (21, 22).

A key distinction between AD early changes and normal aging lies in specificity and progression: AD presents with bilateral asymmetric, progressive atrophy accompanied by entorhinal cortex involvement, closely linked to Tau/amyloid deposition; normal aging shows mild, symmetric, slow atrophy without cognitive decline (Figure 2). Artificial intelligence, especially deep learning models such as convolutional neural networks, has revolutionized the ability to identify these subtle morphological changes. CNNs excel at extracting hierarchical features directly from raw MRI data, enabling automated and precise detection of hippocampal atrophy and cortical thinning without extensive manual preprocessing. Recent studies demonstrate that AI models achieve high classification accuracy in distinguishing early AD from normal aging and mild cognitive impairment, with accuracies often exceeding 90%. Notably, AI’s core advantage in early diagnosis is capturing microstructural variations invisible to the human eye, enabling detection 2–3 years earlier than conventional clinical assessment. These models can capture microstructural variations that may be imperceptible to human observers, facilitating early recognition of AD pathology (18, 23).

**Figure 2:**
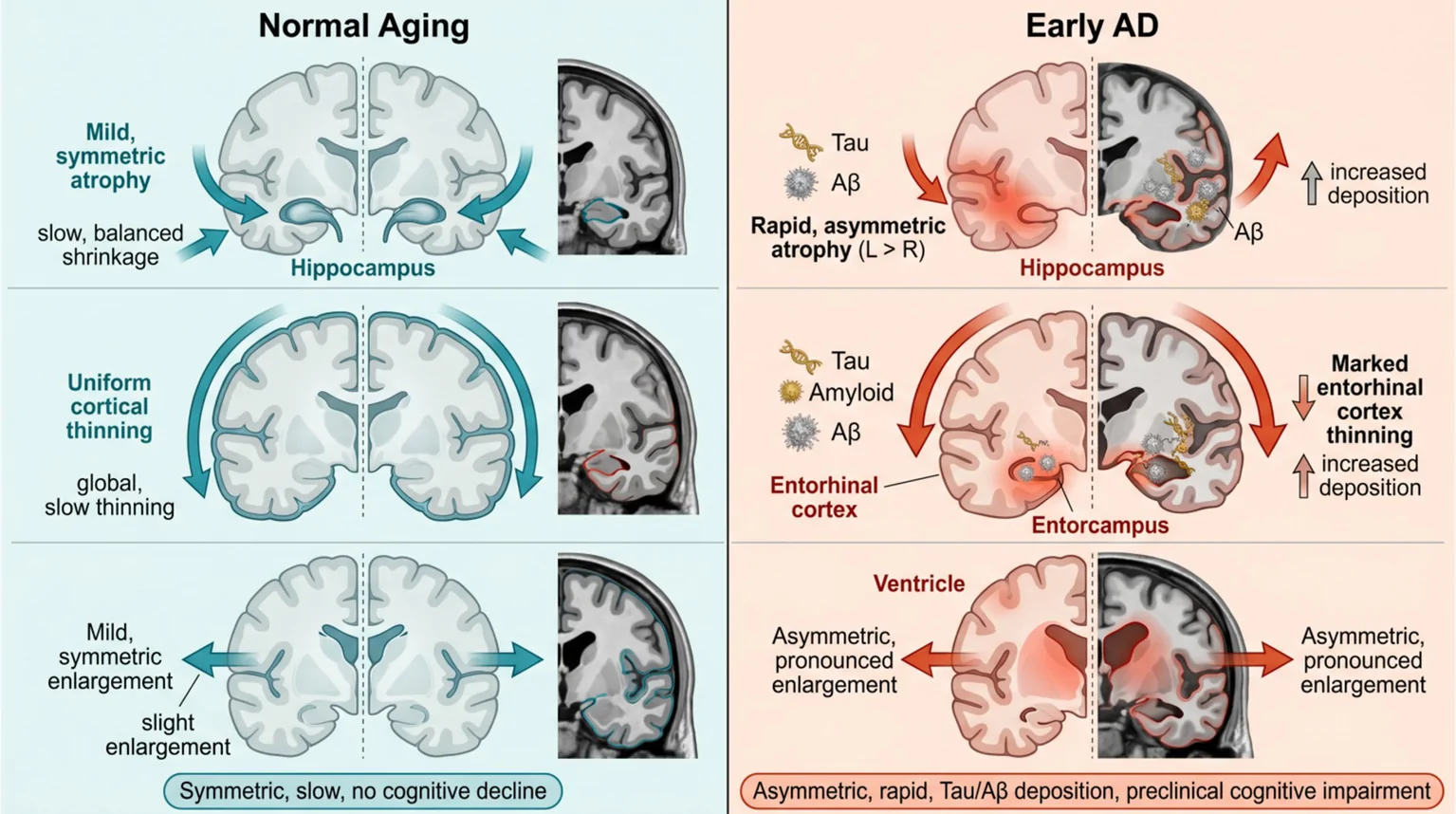
Comparative imaging features of early Alzheimer’s disease (AD) versus normal aging on structural MRI. Schematic conceptual diagrams are displayed side-by-side with representative de-identified real structural MRI slices derived from public open datasets. Left panel: Normal aging shows mild, symmetric hippocampal atrophy, uniform cortical thinning, and slight ventricular enlargement without cognitive decline. Right panel: Early AD exhibits asymmetric, progressive hippocampal atrophy (predominantly left), marked entorhinal cortex thinning, and asymmetric ventricular enlargement, closely associated with tau/amyloid deposition and preclinical cognitive impairment.

Advanced AI frameworks integrate multimodal MRI data and leverage sophisticated architectures such as video vision transformers and ensemble U-Net models to enhance sensitivity to early pathological changes. For instance, video vision transformers treat MRI volumes as sequential data, capturing temporal and spatial dependencies across slices, which improves detection of early hippocampal and cortical alterations. Ensemble approaches combining multiple segmentation networks generate voxel-level disease severity maps, localizing abnormal brain regions with high precision. These innovations contribute to improved classification performance of AI-based AD diagnosis frameworks (16, 24).

Moreover, explainable AI (XAI) techniques have been incorporated to elucidate the decision-making processes of deep learning models, addressing the “black box” challenge. By highlighting key imaging features such as hippocampal atrophy and cortical thinning that drive classification, XAI enhances clinical trust and facilitates integration into diagnostic workflows. The NeuroFusion-ADNet, a multimodal deep learning framework based on convolutional neural networks and attention mechanisms, has shown excellent accuracy while providing interpretable outputs, enabling clinicians to understand and verify AI predictions in early AD diagnosis (25, 26).

In summary, AI-driven analysis of MRI enables the detection of early AD pathological changes, primarily hippocampal atrophy and cortical thinning, with high accuracy and interpretability. These advances hold promise for clinical application in early screening and intervention, potentially altering the disease trajectory through timely therapeutic strategies (Figure 3 illustrates the hierarchical technical workflow for detecting early-stage AD pathological alterations using AI-driven MRI analysis).

**Figure 3:**
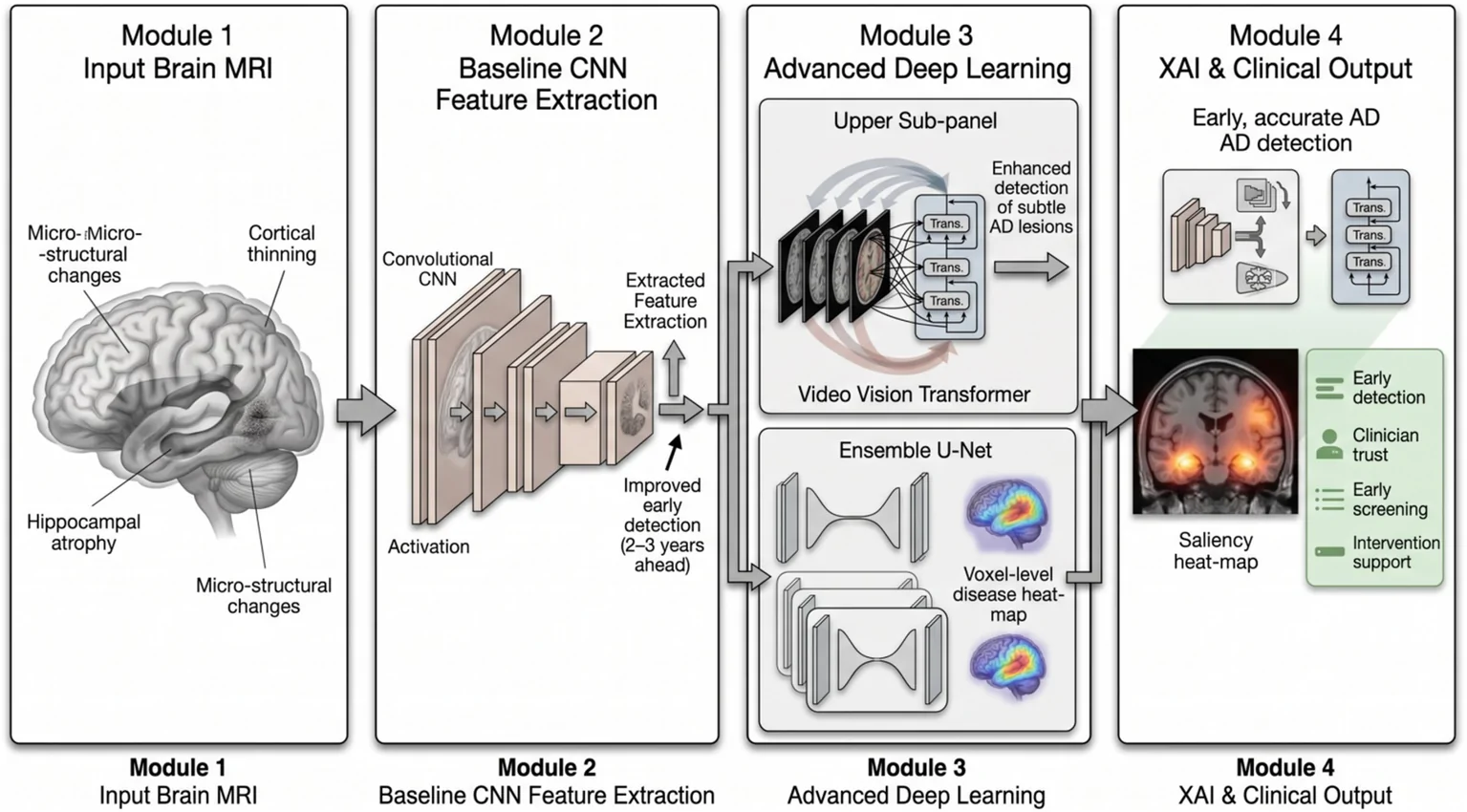
Schematic workflow illustrating how artificial intelligence identifies subtle early pathological alterations of Alzheimer’s disease from brain MRI. mStarting from raw volumetric brain MRI input containing subtle, often human-invisible pathological changes including hippocampal atrophy and cortical thinning, convolutional neural networks (CNNs) perform end-to-end hierarchical feature extraction to detect early AD-related morphological alterations, enabling disease identification approximately 2–3 years ahead of conventional clinical assessment (18, 23). Advanced deep-learning architectures, including video vision transformers and ensemble U-Net models, further improve sensitivity for early lesions: video vision transformers capture cross-slice spatial–temporal dependencies within MRI volumes, while ensemble U-Net frameworks generate voxel-level disease severity maps for precise localization of abnormal brain regions (16, 24). Incorporation of explainable artificial intelligence (XAI) mitigates the “black-box” limitation of deep-learning models by highlighting disease-relevant imaging features such as hippocampal atrophy and cortical thinning. The multimodal attention-based framework NeuroFusion-ADNet serves as a representative example delivering both competitive diagnostic accuracy and interpretable outputs (25, 26). The whole AI pipeline supports early-phase AD screening and facilitates timely therapeutic intervention, with potential to modify disease progression trajectories. AD, Alzheimer’s disease; CNN, convolutional neural network; XAI, explainable artificial intelligence.

### 3.2 Cognitive function score prediction model

Machine learning methods leveraging MRI-derived imaging features have been increasingly applied to predict cognitive function scores such as the Mini-Mental State Examination (MMSE) in AD. These approaches utilize structural and functional brain changes captured by MRI to model the progression of cognitive decline. For example, neural network models integrating brain morphometric parameters and glycemic variability indices have demonstrated high predictive accuracy for cognitive impairment in diabetes-related cognitive decline, achieving up to 98.5% accuracy by combining MRI volumetric data with clinical metrics (27). This illustrates the potential of combining imaging biomarkers with clinical variables to enhance prediction of cognitive scores.

Deep learning frameworks, particularly convolutional neural networks and vision transformers, have been developed to extract hierarchical features from MRI volumes for cognitive status classification. A recent study introduced a video vision transformer model that treats MRI volumes as sequential data, capturing spatial and temporal dependencies to classify normal cognition, mild cognitive impairment, and AD with an accuracy of 98.6% (24). Such models provide fine-grained representations of brain structural changes that correlate with cognitive decline, enabling accurate prediction of cognitive function scores and disease stages.

Ensemble learning approaches combining multiple deep learning models have also shown promise in predicting AD progression and cognitive decline. For instance, an optimized ensemble of long short-term memory (LSTM) networks trained on multivariate longitudinal data, including MRI features and cognitive scores, achieved improved accuracy over individual models, reaching above 82% in progression detection (28). These models capture temporal dynamics of cognitive decline, which are critical for predicting future cognitive function scores.

The predictive accuracy of these models is further enhanced by multimodal data integration. Combining MRI with other biomarkers such as PET imaging, genetic data, and clinical assessments improves the robustness of cognitive function prediction. Studies have demonstrated that multimodal deep learning frameworks outperform single-modality models, achieving classification accuracies exceeding 90% in differentiating AD stages and predicting cognitive decline (15, 29). This suggests that MRI-based features, when fused with complementary data, provide a comprehensive basis for cognitive function score prediction.

It should be emphasized that high accuracy in model results must be interpreted with caution, and all key performance indicators need to be qualified by research conditions: e.g., “98.6% accuracy was achieved in the ADNI dataset (*n* = 968) with internal validation at baseline”—high accuracy does not equate to clinical availability, and sensitivity, specificity, positive/negative predictive values must be prioritized, especially for MCI-to-AD prediction where false positives carry significant clinical risks. The clinical utility of these predictive models lies in their ability to support early diagnosis and personalized treatment planning. Accurate prediction of cognitive scores from MRI enables clinicians to monitor disease progression objectively and tailor interventions accordingly. Moreover, explainable AI techniques applied to these models enhance interpretability, fostering trust and facilitating clinical decision-making (30). Overall, MRI-driven machine learning models for cognitive function score prediction represent a valuable tool in the management of AD and related dementias.

### 3.3 Mild cognitive impairment patient risk assessment

Artificial intelligence models have shown promising capabilities in distinguishing mild cognitive impairment patients who are at high risk of progressing to AD. Deep learning approaches, particularly convolutional neural networks and graph neural networks (GNNs), have been employed to analyze resting-state functional MRI (rs-fMRI) data to extract spatiotemporal features that differentiate MCI from normal controls and AD patients. For instance, a dynamic spatiotemporal graph neural network model effectively captured both temporal and spatial brain connectivity patterns, achieving classification accuracy of 83.78% among AD, MCI, and normal controls, indicating its potential for risk stratification in MCI patients (31). Similarly, a zoom-in neural network deep learning model using rs-fMRI voxel time series data achieved 72.7% accuracy in classifying MCI versus AD and normal controls, highlighting the utility of region-specific functional features in risk assessment (32).

The sensitivity and specificity of different AI algorithms vary depending on the imaging modalities and features used. For example, a 3D convolutional neural network combining rs-fMRI and clinical data predicted conversion from MCI to AD with an accuracy of 87.6%, sensitivity of 75.58%, and specificity of 92.57%, demonstrating improved performance when integrating multimodal data (33). In contrast, FDG-PET imaging combined with deep learning yielded higher accuracy (88.02%) for MCI versus cognitively normal classification compared to MRI-based models, which achieved up to 76.86% accuracy, suggesting that PET may provide complementary metabolic information enhancing risk prediction (34). These findings underscore the importance of selecting appropriate algorithms and multimodal inputs to optimize predictive performance (Table 2 summarizes the complementary value of different MRI modalities in MCI risk assessment; Figure 4).

**Table 2:** Complementary value of MRI modalities for MCI-to-AD risk prediction.

Modality	Core features	Advantages	Limitations	Representative application scenario
Structural MRI (sMRI)	Hippocampal volume, cortical thickness	High specificity, early structural atrophy detection	Insensitive to pre-structural functional changes	Quantify hippocampal atrophy for baseline risk evaluation
rs-fMRI	Functional connectivity, network dynamics	Detects early network damage, pre-structural changes	Low spatial resolution, susceptible to noise	Identify default-mode network disruption in preclinical AD
DTI	White matter integrity, fractional anisotropy	Reflects axonal degeneration, complements structural info	Limited sensitivity to early cognitive decline	Evaluate white-matter tract injury in prodromal MCI

MCI, mild cognitive impairment; AD, Alzheimer’s disease; rs-fMRI, resting-state functional MRI; DTI, diffusion tensor imaging.

**Figure 4:**
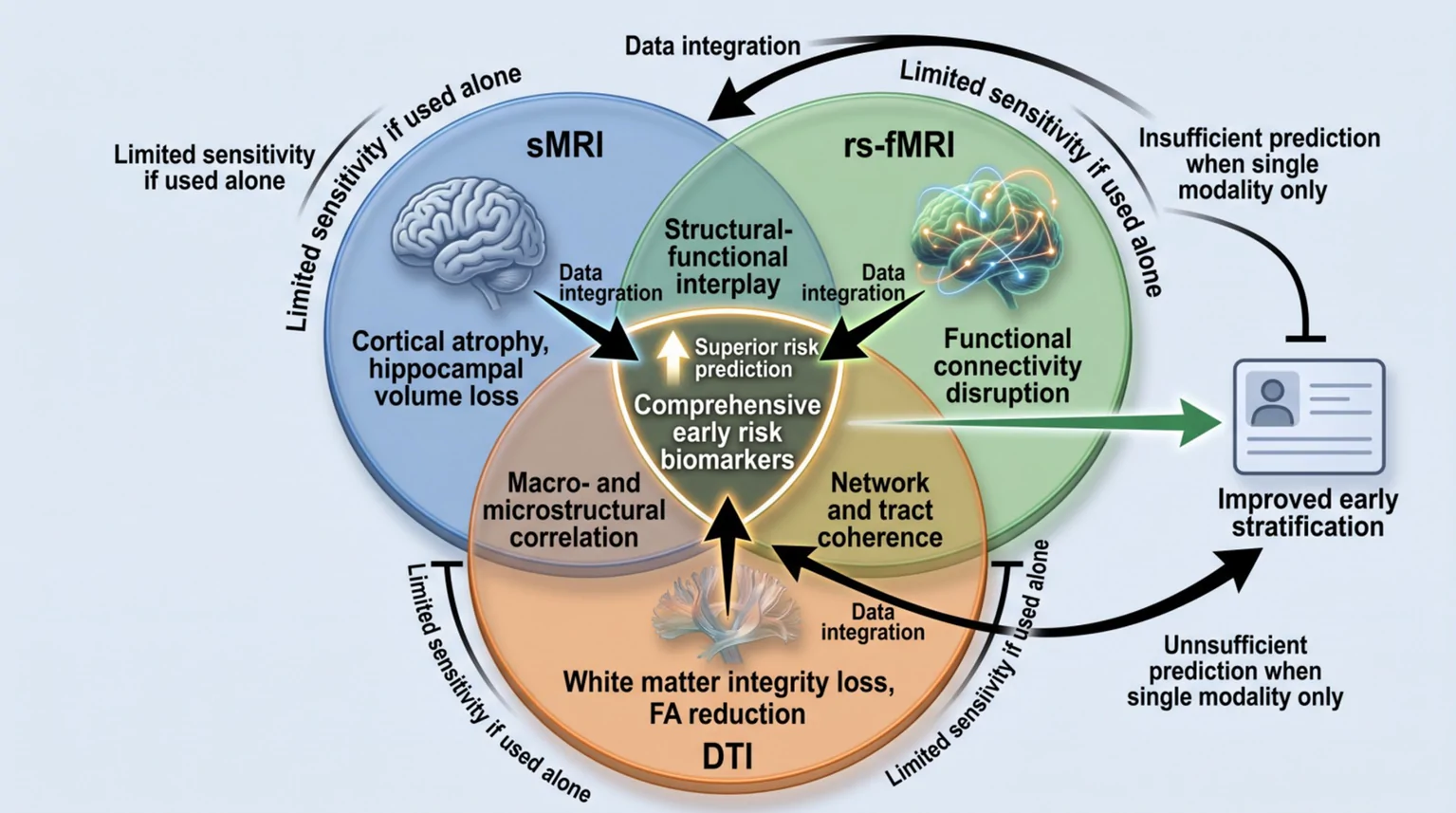
FComplementary value of multimodal MRI for MCI-to-AD risk prediction. The core message is highlighted: multimodal data integration achieves better early-risk-stratification performance than any single-modality MRI analysis. This Venn-style schematic demonstrates unique and overlapping pathological information captured by structural MRI (sMRI), resting-state fMRI (rs-fMRI), and diffusion tensor imaging (DTI). Each modality provides distinct biomarkers reflecting different dimensions of AD-related neurodegeneration; limitations for single-modality detection are explicitly annotated.

Of note, most published studies only report overall classification accuracy. The lack of routinely reported sensitivity and specificity creates risks for over-optimistic interpretation of model performance; high accuracy from internal validation does not guarantee reliable performance for unseen real-world cohorts. Such methodological limitations should be kept in mind when comparing results across different studies.

Advanced AI frameworks incorporating dynamic functional connectivity (dFC) and attention mechanisms have further enhanced risk prediction in MCI. A convolutional recurrent neural network (CRNN) model leveraging dFC networks from rs-fMRI captured sequential brain network dynamics and outperformed traditional machine learning methods in classifying AD, MCI, and normal controls, emphasizing the value of temporal information in risk assessment (35). Moreover, transformer-based architectures combined with graph convolutional networks (GCNs) have improved brain age prediction accuracy, enabling identification of early neurodegenerative changes and contributing to the intervention and management of AD (36). These models provide more nuanced representations of brain network alterations associated with cognitive decline.

Explainable AI techniques have been applied to enhance interpretability and clinical trust in AI-based risk assessments. For example, models using fMRI complexity measures such as sample entropy matched or exceeded tau PET performance in classifying MCI from normal cognition, with the dorsal attention network reported to be critical for classification (37, 38). Similarly, graph convolutional networks quantifying structure–function discrepancies in brain connectivity demonstrated high accuracy (88%) in classifying subjective cognitive impairment from AD dementia and identified neurologically relevant cortical regions, supporting their use as biomarkers for AD classification (39). These interpretable models facilitate understanding of underlying pathophysiology and may guide personalized interventions.

In summary, AI-driven MRI analyses for MCI risk assessment leverage diverse algorithms ranging from CNNs and GNNs to transformer-based models, often integrating multimodal imaging and clinical data to enhance sensitivity and specificity. Dynamic functional connectivity and brain complexity metrics provide valuable features for early detection of individuals likely to progress to AD. Explainable AI approaches further support clinical applicability by elucidating brain regions and networks implicated in disease progression. Continued refinement and validation of these models hold promise for improving prognostic accuracy and enabling timely therapeutic strategies in MCI populations.

## 4 AI-assisted MRI in the monitoring and prognostic evaluation of AD progression

### 4.1 Tracking of imaging biomarkers during disease progression

Artificial intelligence technologies have increasingly been applied to longitudinal MRI datasets to identify and track pathological progression in AD. Machine learning models, such as Random Forest and convolutional neural networks, have demonstrated the capacity to analyze complex temporal patterns in neuroimaging data, enabling classification of disease trajectories from healthy aging through mild cognitive impairment to AD dementia. For example, Random Forest models applied to the ADNI cohort achieved high sensitivity and specificity in distinguishing stable, convertible, and reverse progression groups, highlighting key neuroimaging biomarkers including temporal amyloid uptake variations and amyloid biomarkers associated with para-hippocampal regions (40). These AI-driven approaches facilitate dynamic monitoring of disease evolution beyond static imaging snapshots.

The relationship between imaging biomarkers and clinical symptom progression is critical for understanding AD pathophysiology. Studies have shown that baseline brain volumes, particularly of the hippocampus, thalamus, and lateral ventricles, serve as robust predictors of cognitive decline and MCI conversion, outperforming short-term atrophy rates. This confirms that structural brain reserve (reflected by baseline volumetric MRI measures) underpins resilience to neurodegeneration, with stronger correlations to memory and executive function impairment than annual atrophy rates. This suggests that structural brain reserve, as reflected by volumetric MRI measures, underpins resilience against neurodegeneration and correlates with memory and executive function impairments (41). Such findings emphasize the prognostic value of early imaging markers in anticipating clinical deterioration.

Visual rating scales applied to MRI, such as the Comprehensive Visual Rating Scale (CVRS), have also been validated for predicting progression from prodromal AD to dementia. Higher baseline CVRS scores, reflecting greater structural brain changes, independently predict conversion risk over longitudinal follow-up, underscoring the clinical relevance of semi-quantitative imaging assessments in disease tracking (42). This approach complements AI-based quantitative analyses by providing interpretable metrics for clinicians.

Recent advances in portable low-field MRI combined with machine learning pipelines enable morphometric quantification of brain structures and white matter hyperintensities in outpatient settings. These developments increase accessibility to longitudinal imaging assessments, potentially allowing more frequent monitoring of AD progression in diverse clinical environments (43). The integration of AI enhances the accuracy and automation of biomarker extraction from such data (To guide clinical practice, Table 3 summarizes the core MRI imaging biomarkers for AD progression monitoring).

**Table 3:** Core MRI imaging biomarkers for AD progression monitoring.

Biomarker category	Key Indicators	Prognostic value
Structural volume	Hippocampal volume, entorhinal cortex thickness	Strongest predictor of 2–5-year cognitive decline
Atrophy rate	Temporal/parietal cortex atrophy speed	Reflects disease activity, suitable for late-stage monitoring
Ventricular change	Lateral ventricle enlargement rate	Simple, reliable, correlates with global brain atrophy
Functional connectivity	Default mode network connectivity strength	Early marker, appears prior to measurable structural atrophy

AD, Alzheimer’s disease.

Overall, AI-driven analysis of longitudinal MRI data offers powerful tools for tracking AD progression by identifying imaging biomarkers that correlate with clinical decline. These methods support early detection, prognosis, and personalized disease management by linking structural and functional brain changes to symptom trajectories. The combination of volumetric, visual rating, and advanced machine learning techniques provides a comprehensive framework for monitoring neurodegeneration over time.

### 4.2 Construction and validation of the prognosis model

The construction of prognostic models for AD has increasingly leveraged longitudinal MRI data combined with deep learning techniques. A notable approach involves using multi-timepoint MRI scans to capture the dynamic progression of brain changes, which are then modeled through advanced neural networks. For instance, a two-stage framework employing an ensemble of 125 U-Nets generates voxel-level disease severity maps, which are subsequently analyzed via graph convolutional neural networks to classify and predict AD progression, demonstrating competitive performance and improved generalizability across datasets (16). This highlights the potential of integrating spatial and temporal MRI features for robust prognosis.

Deep learning models, particularly convolutional neural networks and their variants, have shown strong capabilities in extracting hierarchical features from raw MRI data, facilitating the prediction of cognitive decline speed and survival outcomes. Hybrid models combining CNNs with recurrent architectures like long short-term memory networks effectively capture temporal dependencies in longitudinal data, enhancing prognostic accuracy. For example, a hybrid framework integrating LSTM for clinical data and ResNet50/MobileNetV2-based feature extraction from MRI achieved 96.19% accuracy on ADNI datasets and 99.82% accuracy on NACC datasets, underscoring the value of multimodal temporal modeling (44). Such models enable early identification of patients at higher risk of rapid progression.

The use of longitudinal whole-brain 3D MRI data combined with neurocognitive tests has further improved prognostic predictions. A two-stage deep learning framework first extracts latent features from single-timepoint MRI scans using 3D CNNs, then concatenates these with longitudinal cognitive data for risk prediction of conversion from mild cognitive impairment to AD within 4 years. This approach achieved a balanced accuracy of 83.4%, significantly outperforming single-modality or single-timepoint models (45). The explainability of these models is enhanced by heatmaps highlighting brain regions critical for prognosis, facilitating clinical interpretability.

Ensemble learning methods that combine multiple machine learning classifiers have also been applied to MRI data for AD prognosis. These ensemble classifiers outperform individual models by 3–5% in accuracy, reaching up to 96.5% in classification tasks distinguishing AD, MCI, and cognitively normal subjects (46). Such methods are particularly advantageous when dealing with limited labeled data, as they reduce overfitting risks and improve robustness. Moreover, integrating advanced preprocessing techniques like skull stripping and intensity normalization enhances feature quality for prognostic modeling (47).

For clinical translation, prognosis models must prioritize interpretability and operability: Grad-CAM heatmaps to mark key prognostic brain regions, risk stratification (high/medium/low risk) output, and seamless integration with clinical scales (e.g., MMSE, CDR) are core requirements for clinician acceptance. The integration of deep learning with advanced feature extraction and fusion techniques has led to models capable of predicting cognitive decline trajectories and survival times. For example, combining deep features from CNNs with texture-based shearlet coefficients and discriminant correlation analysis yielded classification accuracies of 94.46% at baseline and 95.44% at 24 months, with an accuracy of 92.97% at 12 months, for early diagnosis of AD (47). These results emphasize the importance of multimodal feature integration and longitudinal data in constructing accurate and clinically useful prognostic models for AD.

### 4.3 Imaging omics and multimodal data fusion

The integration of MRI with other modalities such as PET and genomics through artificial intelligence techniques has become a pivotal approach in enhancing disease characterization and prognostication. AI-driven multimodal data fusion methods leverage complementary information from diverse sources, enabling a more comprehensive understanding of pathological processes than single-modality analyses. For instance, combining radiomic features extracted from MRI with clinical and molecular data has shown promise in oncology for improved tumor subtyping and risk stratification. These AI models utilize machine learning (ML) and deep learning algorithms to extract and integrate high-dimensional features, facilitating personalized assessment and clinical decision-making (48).

Mechanistically, multimodal fusion enhances prognostic accuracy by capturing the multidimensional heterogeneity of diseases. Radiomics quantifies imaging phenotypes, while genomics and other omics provide molecular insights, and clinical data contextualize patient-specific factors. AI models can identify latent correlations and synergistic patterns across these data types, which are often imperceptible to human observers. For example, in hepatocellular carcinoma, tensor fusion techniques integrating MRI radiomics and histopathology have improved early recurrence prediction by effectively handling multi-scale and multi-dimensional data (49). Similarly, logistic regression-based fusion models combining clinical and imaging features have demonstrated superior performance in differentiating benign from malignant tumors compared to unimodal models (50) (Table 4 compares the advantages and limitations of common multimodal fusion strategies).

**Table 4:** Comparison of multimodal fusion strategies for AD-MRI.

Fusion type	Implementation method	Advantages	Limitations
Early fusion	Input-layer concatenation of multimodal raw data	Strong information complementarity, supports end-to-end learning	High data dimensionality, difficult model training, poor interpretability
Late fusion	Independent model training for each modality + fusion of output prediction results	Simple implementation, stable performance, low training cost, relatively interpretable	Weak capacity to capture cross-modal feature interaction

AD, Alzheimer’s disease; MRI, magnetic resonance imaging.

Recent studies also emphasize the role of advanced AI frameworks that incorporate explainability and robust validation to facilitate clinical translation. Explainable AI models integrating MRI radiomics, natural language processing of radiology reports, and clinical variables have improved preoperative risk assessments in cancers such as cervical cancer, highlighting the importance of multimodal fusion for actionable insights (51). Moreover, frameworks like MONAI provide domain-specific tools optimized for multimodal data handling, enhancing personalized multimodal risk assessment in orthopedics (52). These advances underscore the growing recognition that multimodal fusion is essential for precision medicine.

The integration of MRI with PET and genomic data through AI not only improves diagnostic and prognostic accuracy but also supports dynamic treatment monitoring and outcome prediction. For example, in pulmonary tuberculosis, multimodal AI models combining imaging and genomic data have shown potential in individualized outcome prediction and treatment response monitoring (53). Similarly, in prostate cancer, AI applied to multimodal MRI imaging has enhanced lesion detection and risk stratification (54). These findings collectively highlight that multimodal data fusion powered by AI represents a transformative approach to improving clinical care.

In summary, AI-driven multimodal data fusion combining MRI with PET, genomics, and clinical data harnesses complementary information to enhance disease characterization and prognostication. By integrating heterogeneous data sources, these approaches improve predictive accuracy and support personalized medicine. Continued development of explainable, robust, and clinically validated fusion models is critical to translating these advances into routine clinical practice.

## 5 Challenges and solutions in clinical applications

### 5.1 Data quality and sample heterogeneity issues

Magnetic resonance imaging data quality and sample heterogeneity present significant challenges in AD research, particularly when artificial intelligence methods are applied. Variability in MRI acquisition protocols across different scanners and institutions leads to differences in image contrast, resolution, and noise levels, which can adversely affect the performance and generalizability of AI models. For example, studies have highlighted that differences in scanning parameters and hardware contribute to domain shifts that complicate model training and testing across datasets (55). Such heterogeneity can result in inconsistent feature representations, making it difficult to develop robust diagnostic algorithms.

To address these issues, data preprocessing and standardization techniques have been widely adopted. Image harmonization methods, such as those based on adversarial generative networks, have been shown to reduce scanner-dependent effects by adjusting images to a common domain, thereby improving classification accuracy for AD (55). Additionally, spatial normalization techniques using deep learning have been developed to align images without requiring paired MRI scans, enhancing quantification consistency across subjects and scanners (56). These preprocessing steps are crucial for mitigating inter-site variability and enabling more reliable AI-driven analyses.

Deep learning architectures often incorporate data augmentation and transfer learning strategies to cope with limited and heterogeneous datasets. Transfer learning, which leverages pretrained convolutional neural networks on large datasets, has demonstrated improved performance in AD classification tasks, especially when training data are scarce or heterogeneous (57, 58). Moreover, multi-task learning frameworks that jointly perform diagnosis and atrophy localization have been proposed to capture both global and local structural information, enhancing robustness against sample heterogeneity (59). These approaches help models generalize better by learning shared representations across diverse data.

Quality assurance of MRI data is another critical aspect influencing AI model reliability. Automated quality assessment using deep learning has been applied to detect artifacts such as motion and low contrast, which degrade image quality and affect downstream analyses (60, 61). Ensuring high-quality input data through such automated pipelines reduces noise and bias introduced by poor-quality images, thereby improving the consistency of AI model outputs. This is particularly important in large-scale multicenter studies where manual quality control is impractical.

Practical solutions to data heterogeneity include: (1) GAN-based image harmonization to unify multi-scanner data distributions; (2) Federated learning to train models across centers without data sharing; (3) Weakly supervised learning to leverage unannotated multi-center data; (4) Adaptive data augmentation to simulate scanner variability. Federated learning represents a promising solution for multi-center AD-MRI research: it enables joint model training without transferring raw sensitive neuroimaging data across sites. Nevertheless, practical barriers remain including heavy communication overhead and performance degradation caused by cross-site scanner heterogeneity.

In summary, the heterogeneity of MRI data due to non-uniform acquisition standards poses a significant challenge for AI applications in AD research. Advanced preprocessing techniques including image harmonization, spatial normalization, and quality assurance, combined with transfer learning and multi-task learning strategies, are essential to mitigate these issues. These methods collectively enhance the robustness and generalizability of AI models, facilitating more accurate and reliable diagnosis and prognosis of AD.

### 5.2 Model generalization capability and clinical applicability

Artificial intelligence models applied to MRI for AD diagnosis face significant challenges in generalizability across diverse populations and imaging devices. Variability in MRI acquisition protocols, scanner types, and demographic heterogeneity can lead to performance degradation when models trained on one dataset are applied to others. For instance, many AI models rely heavily on datasets such as ADNI, which limits diversity and may cause overfitting to specific imaging characteristics or population traits (18). This restricts the clinical applicability of AI models, as real-world settings often involve heterogeneous data sources.

Cross-center validation is a critical strategy to assess and improve model generalization. Studies have emphasized the importance of validating AI models on external datasets from different institutions to ensure robustness. For example, a two-stage framework using an ensemble of 125 U-Nets demonstrated better generalization by grading disease severity voxel-wise and employing graph convolutional networks for classification, showing competitive performance across multiple datasets (16). Such approaches highlight that incorporating diverse training data and multi-stage architectures can enhance model adaptability.

Model fine-tuning and transfer learning are widely adopted to address domain shifts between centers. Transfer learning with convolutional neural networks pretrained on large image datasets or related medical imaging tasks can improve performance on smaller, institution-specific datasets. However, some studies report that training models from scratch on domain-specific data may outperform transfer learning in certain MRI quality assurance tasks, suggesting that the optimal strategy depends on the application and data availability (60). Moreover, data augmentation techniques, including synthetic data generation via quantum generative adversarial networks (QGANs), have been proposed to mitigate limited data issues and improve model robustness (62).

The integration of multimodal data and ensemble learning further enhances clinical applicability. Combining MRI with other biomarkers, such as gene expression data, and fusing features through attention-based mechanisms have shown improved diagnostic accuracy and interpretability (63). Ensemble models that aggregate predictions from multiple architectures can reduce variance and improve stability across different populations. Nevertheless, challenges remain in standardizing data preprocessing, harmonizing imaging protocols, and ensuring explainability to foster clinician trust and facilitate regulatory approval.

Many existing AI models for AD-MRI are validated on retrospective public datasets. Prospective multi-center cohort validation is urgently needed for clinical translation. Substantial performance drop is frequently observed when models are applied to external independent cohorts, highlighting the necessity of strict external validation. Reproducibility challenges persist: many published studies lack publicly available source code and complete preprocessing pipelines. Additional practical barriers include seamless integration into existing radiology reporting workflows, regulatory medical-AI device certification requirements, and cost-effectiveness considerations for hardware deployment, especially within primary-care and resource-limited clinical settings.

Five actionable strategies to enhance model generalization: (1) Train on multi-center, multi-device, multi-ethnic datasets (prioritize Asian populations to complement ADNI’s Western bias); (2) Mandate internal + ≥2 external independent validations; (3) Adopt domain adaptation/meta-learning to reduce inter-center differences; (4) Lightweight models for edge deployment in primary care; (5) Co-design models with clinicians to integrate clinical prior knowledge. In summary, AI model generalization and clinical applicability depend on rigorous cross-center validation, effective domain adaptation strategies, and incorporation of diverse multimodal data. Continued efforts in dataset sharing, standardized evaluation, and explainable AI are essential to translate AI-driven MRI diagnostics for AD into routine clinical practice.

### 5.3 Ethical, privacy, and regulatory issues

The protection of patient data privacy is paramount in the application of artificial intelligence in AD diagnosis using MRI. MRI datasets often contain sensitive personal health information, and the aggregation of large-scale imaging data for AI training raises concerns about unauthorized access and misuse. Ensuring compliance with data protection regulations such as GDPR and HIPAA is essential to safeguard patient confidentiality. Moreover, anonymization and secure data storage protocols must be rigorously implemented to prevent re-identification risks, especially given the high dimensionality and uniqueness of neuroimaging data.

AI-assisted diagnostic tools face significant ethical and regulatory challenges in clinical settings. The opacity of many deep learning models, often described as “black boxes,” complicates the interpretability of AI decisions, which is critical for clinical trust and acceptance. Multiple explainable artificial intelligence techniques are available for neuroimaging studies, each with inherent advantages and limitations. Grad-CAM generates intuitive activation heat-maps highlighting image regions driving model decisions, but offers limited quantitative biological interpretability. Layer-wise relevance propagation (LRP) provides voxel-wise relevance scoring yet brings high computational cost for 3D volumetric brain MRI. SHAP can deliver consistent feature-importance quantification, but imposes heavy computing burden for high-dimensional whole-brain data. Attention-map visualization for Transformer and graph-neural-network models can reflect inter-region relationships, yet may produce noisy activations without clear neuropathological meaning. Major unsolved challenges include the absence of standardized metrics to evaluate explanation quality, as well as difficulties validating whether XAI outputs truly correspond to established AD neuropathological findings. However, the integration of these tools into clinical workflows requires adherence to regulatory standards that ensure safety, efficacy, and accountability (30, 64).

Regulatory frameworks must address the validation and generalizability of AI models across diverse populations and imaging protocols. Many AI studies rely heavily on datasets like ADNI, which may limit demographic and scanner variability, potentially biasing model performance. This raises ethical concerns about equitable healthcare delivery and the risk of misdiagnosis in underrepresented groups. AI for MRI diagnosis of AD faces challenges including methodological heterogeneity, overfitting, and limited dataset diversity; multimodal deep learning for early AD diagnosis additionally faces challenges including insufficient datasets, high computational cost, and lack of clinical integration.

The use of AI in AD diagnosis also prompts considerations regarding informed consent and patient autonomy. Patients should be made aware of how their imaging data will be used, including potential secondary uses for AI training and research. When machine learning and deep learning techniques are applied to AD diagnosis, they support rather than replace clinical assessment by medical professionals (29).

Core countermeasures for ethics, privacy, and regulation: (1) Privacy: Federated learning, differential privacy, data anonymization, blockchain-based data management; (2) Explainability: Mandatory XAI modules, traceable decision-making, clinician final review mechanism; (3) Fairness: Enroll diverse age/gender/ethnicity/education groups to avoid demographic bias; (4) Regulation: Comply with medical device approval, conduct clinical trials and real-world evidence studies. In summary, the ethical, privacy, and regulatory landscape surrounding AI-driven MRI diagnostics in AD is complex and evolving. Protecting patient data privacy, ensuring model transparency and fairness, complying with regulatory standards, and maintaining ethical clinical practices are critical to the responsible advancement and clinical adoption of AI technologies in this field.

## 6 Latest research findings and technological innovations

### 6.1 AI model training based on a large-scale multi-center database

The utilization of large-scale, multicenter databases such as the AD Neuroimaging Initiative (ADNI) offers significant advantages for developing AI models in AD research. These publicly available datasets provide standardized, well-curated magnetic resonance imaging data from diverse populations, enabling robust training and validation of deep learning algorithms. For instance, employing ADNI data allows models to capture a wide spectrum of disease presentations and imaging variations, which enhances generalizability and clinical applicability. The availability of such extensive datasets facilitates the extraction of subtle imaging features associated with AD progression, which might be overlooked in smaller cohorts.

It is critical to recognize the limitations of mainstream databases: ADNI/AIBL/OASIS feature high data quality and precise annotation but are dominated by Western populations, with insufficient Asian cohorts, single-device bias, and limited long-term follow-up—restricting model performance in non-Western populations. In the context of large data volumes, deep learning models have demonstrated marked improvements in performance. For example, a study using a densely connected convolutional neural network with connection-wise attention mechanisms trained on 968 subjects from ADNI achieved classification accuracies of 97.35% for AD versus healthy controls and 78.79% for distinguishing mild cognitive impairment converters from non-converters. This highlights how leveraging large single-center datasets can enhance the detection of early and prodromal stages of AD by enabling models to learn complex, multiscale features from whole-brain MRI (65). Similarly, attention-guided hybrid networks trained on multiple public datasets including ADNI have shown superior performance in both AD diagnosis and MCI conversion prediction, underscoring the value of large-scale, heterogeneous data for model robustness (66).

Transfer learning techniques further capitalize on large databases by enabling models pretrained on extensive datasets to be fine-tuned for specific tasks or smaller datasets. This approach addresses challenges related to limited labeled data and domain shifts across imaging protocols. For instance, transfer learning applied to ADNI MRI data using pretrained convolutional neural networks such as ResNet-101 achieved a high accuracy of 98.37% in multiclass classification of AD, MCI, and normal controls. This demonstrates that large-scale databases provide training data for transfer learning studies on AD classification and facilitate the development of transfer learning applications for magnetic resonance brain imaging.

Moreover, harmonization of MRI data from multiple centers and scanners is crucial to mitigate variability and improve AI model performance. An attention-guided generative adversarial network (AG-GAN) was employed to harmonize images from ADNI, AIBL, and OASIS datasets, leading to significantly improved AD classification accuracy compared to non-harmonized data. This indicates that large multicenter databases, when combined with advanced AI-based harmonization techniques, can overcome inter-site heterogeneity and enhance diagnostic precision (55). The integration of such harmonized large datasets is pivotal for developing AI models with strong generalizability and clinical utility.

In summary, large-scale multicenter databases like ADNI provide a rich resource for training AI models in AD research. They enable improved model accuracy, robustness, and generalizability through diverse and standardized imaging data. Transfer learning and data harmonization techniques further augment these benefits, facilitating early diagnosis and prognosis prediction in AD. The continued expansion and sharing of such databases will be instrumental in advancing AI-driven neuroimaging applications for dementia.

### 6.2 Emerging deep learning architectures and self-supervised learning

Emerging deep learning architectures such as Transformers and graph neural networks have recently been applied to MRI analysis for AD diagnosis, offering novel capabilities beyond traditional convolutional neural networks. For instance, vision transformer architectures have demonstrated strong performance in classifying AD stages from MRI scans by capturing long-range dependencies and global contextual information, which are difficult for CNNs to model effectively. A study employing a vision transformer combined with CNN elements achieved detection accuracies up to 89% for AD versus control groups, highlighting the potential of these architectures in extracting discriminative features from complex brain images (4). This approach leverages the transformer’s ability to model spatial relationships across the entire image, which is critical given the diffuse and heterogeneous nature of AD-related brain changes.

Graph neural networks represent another promising direction, as they can model the brain as a graph structure, capturing relationships between different brain regions. Spectral graph convolutional neural networks (SGCNNs) have been used to analyze functional brain changes in MRI, achieving classification accuracies around 95% for AD diagnosis. Spectral graph convolutional neural network models have been developed for AD diagnosis (67). By focusing on the brain’s network topology, GNNs provide a biologically meaningful framework that aligns with the known pathophysiology of AD, where network disruptions precede overt atrophy (Table 5 compares the core advantages and applicable scenarios of mainstream deep learning architectures).

**Table 5:** Comparison of deep learning architectures for AD-MRI analysis.

Architecture	Core strengths	Applicable scenarios	Limitations
CNN	Efficient local feature extraction, moderate computational overhead	Structural MRI, brain tissue segmentation	Limited capacity for modelling global long-range brain dependencies
Transformer	Global contextual modelling, capture long-range spatial dependency	3D volumetric MRI, functional MRI analysis	High computational cost, requires large volume of annotated training data
GNN	Biologically plausible brain network topology modelling	rs-fMRI, functional connectivity analysis	Performance heavily depends on predefined graph construction strategy

AD, Alzheimer’s disease; CNN, convolutional neural network; GNN, graph neural network; rs-fMRI, resting-state functional MRI.

Self-supervised learning (SSL) methods have gained attention for their ability to reduce reliance on large labeled datasets, which are scarce and costly to obtain in medical imaging. In neuroimaging AD research, mainstream self-supervised pretext tasks can be divided into three categories: image reconstruction, contrastive learning and masked volume modelling. Reconstruction-type SSL tasks require networks to restore corrupted or incomplete brain MRI volumes, forcing models to learn intrinsic anatomical prior knowledge of brain structures. Contrastive selfsupervised learning generates multiple augmented views from one original MRI sample and guides the model to pull representations of homologous samples closer while pushing heterogeneous samples apart. Masked volume modelling randomly masks partial voxels of 3D-MRI volumes and lets networks predict missing content, which fits well with 3D volumetric brain data. SSL pre-trained weights can be transferred to downstream AD classification, segmentation and MCI conversion prediction tasks, greatly alleviating the shortage of annotated neuroimaging samples. Even so, selfsupervised learning still has bottlenecks: the quality of pre-training heavily depends on the diversity of unlabeled datasets; pretext task design lacks unified optimization standards for brain MRI, and the biological interpretability of SSL-learned representations remains insufficient.

Graph neural networks also contain multiple sub-branches for AD-MRI analysis. Spatial graph convolutional networks aggregate features from physically adjacent brain regions, while spectral graph convolutional networks operate on graph Laplacian matrices to mine frequency-domain brain network signals. Graph attention networks further introduce attention weights to assign different importance to pairwise brain-region connections, which is more suitable for modelling heterogeneous functional connectivity alterations occurring in early AD. This unsupervised learning approach addresses the challenge of limited annotated MRI scans for AD, achieving a classification accuracy of 0.84 even with limited labeled data (68). The combination of SSL with emerging architectures like transformers and GNNs could further enhance feature learning by exploiting both global and relational information in brain images.

Moreover, hybrid models integrating CNNs with attention mechanisms or transformer modules have shown improved accuracy and interpretability. For example, multimodal frameworks combining MRI images with clinical metadata through cross-attention mechanisms have achieved classification accuracies exceeding 98% for multiclass AD diagnosis, demonstrating the benefit of fusing heterogeneous data sources (15). Attention modules help the model focus on the most informative spatial and channel-wise features, enhancing the detection of subtle neurodegenerative changes.

In summary, emerging deep learning architectures such as transformers and graph neural networks, combined with self-supervised learning strategies, offer significant advances in MRI-based AD diagnosis. These models improve feature extraction by capturing complex spatial and relational patterns, reduce dependence on large labeled datasets, and enable integration of multimodal data, collectively enhancing early and accurate detection of AD (Key attributes of representative published AI-MRI studies for AD analysis are summarized in Table 6).

**Table 6:** Summary of representative AI-MRI studies for Alzheimer’s disease analysis.

Study reference	Dataset source	MRI modality	AI architecture	Main evaluation metrics	Strengths	Limitations	Clinical applicability	Generalization capability
Nguyen et al. (16)	Multi-center (ADNI + local cohort)	sMRI	Ensemble U-Net + GCN	Classification accuracy, voxel-level severity map	Ensemble strategy; voxel-wise disease grading; partial interpretability	High computational consumption; limited external multi-ethnic validation	Support auxiliary staging; not suitable for primary-care real-time screening	Moderate; improved via multi-dataset training
Akan et al. (24)	ADNI	sMRI volumetric	Video Vision Transformer	Accuracy 98.6% (NC/MCI/AD)	Captures slice-wise spatio-temporal dependency; excellent volumetric feature mining	High GPU memory demand; trained mainly on Western cohorts	Research-oriented auxiliary diagnosis; heavy hardware requirement	Moderate; needs further domain adaptation for non-ADNI data
An et al. (31)	ADNI	rs-fMRI	Dynamic spatiotemporal GNN	Accuracy 83.78% (NC/MCI/AD)	Model dynamic brain functional connectivity; suitable for pre-structural injury detection	Only overall accuracy reported; lack sensitivity/specificity	MCI risk stratification for research cohorts	Limited; performance drops on heterogeneous scanner data
Ghafoori et al. (33)	ADNI	rs-fMRI + clinical metadata	3D-CNN	Accuracy 87.6%; Sensitivity 75.58%; Specificity 92.57%	Multimodal fusion of imaging and clinical variables; complete set of classification metrics	Relatively small MCI-conversion subset	Binary prediction for MCI-to-AD conversion risk stratification	Moderate; requires external real-world cohort verification
Zhang et al. (65)	ADNI	sMRI	3D densely connected CNN with attention	AD vs. NC acc 97.35%; MCI-converter vs. non-converter acc 78.79%	Attention mechanism; good performance for prodromal AD detection	Ethnicity bias (Western-dominated dataset)	Screening auxiliary tool for research setting	Poor without additional harmonization steps
Lian et al. (66)	ADNI + AIBL	sMRI	Attention-guided hybrid network	Multiclass classification accuracy	Multi-public-dataset training; attention-based interpretable output	Interpretability is post-hoc, not inherent to model	Auxiliary diagnosis for memory clinic	Moderate, performance varies across scanner vendors
Dual-Model AI Framework for AD Diagnosis [Ref-A]	Single-center hospital cohort	sMRI + clinical scale data	Dual-model multimodal network	Binary classification accuracy	Seamlessly combines clinical scale information and MRI imaging features	Single-center source; lack multi-ethnic external validation	Supports clinical-oriented multimodal risk assessment	Low, needs multi-center validation

ADNI, Alzheimer’s disease neuroimaging initiative; AIBL, Australian imaging, biomarker and lifestyle flagship study of aging; NC, cognitively normal controls; GPU, graphics processing unit. Most included studies are mainly trained and validated on Western-origin public datasets, which may bring demographic bias. High internal-dataset accuracy does not guarantee reliable performance in real-world multi-ethnic multi-scanner cohorts.

### 6.3 Real-time MRI image analysis and clinical assistance system

Artificial intelligence-driven real-time MRI image analysis has emerged as a transformative technology, enabling rapid processing and interpretation of dynamic imaging data. Real-time MRI captures continuous image sequences, often at high temporal resolution, generating large datasets that require efficient and automated analysis methods. Deep learning techniques, particularly convolutional neural networks, have been successfully applied to segment anatomical structures and pathological regions in real-time MRI, facilitating immediate clinical decision-making. For example, a fully automated 2D CNN named PrimUNet was developed for segmenting cardiac structures in non-human primates, achieving high accuracy comparable to human experts and enabling strain analyses of individual heartbeats (69). This demonstrates the feasibility of AI-based segmentation in real-time MRI, which is critical for clinical workflows requiring rapid image interpretation.

The design of clinical auxiliary diagnostic systems based on AI integrates multiple components, including image acquisition, preprocessing, segmentation, feature extraction, and classification. These systems leverage deep learning models trained on large annotated datasets to identify disease-specific imaging biomarkers. In AD diagnosis, AI models have been developed to analyze hippocampal atrophy and other structural changes from MRI scans with high accuracy. For instance, transfer learning with pre-trained CNNs such as ResNet-101 achieved classification accuracy up to 98.37% in distinguishing AD, mild cognitive impairment, and normal controls, highlighting the clinical utility of AI-assisted MRI analysis (57). Moreover, attention-guided hybrid networks have been proposed to localize discriminative brain regions and fuse multilevel features, improving diagnostic performance (66). Although originally developed for neuro-oncology tasks, deployable real-time explainable CNN frameworks provide valuable technical reference for constructing AD-oriented clinical auxiliary systems (70). It should be noted that these tumor-focused pipelines still require targeted adaptation for brain atrophy and neurodegeneration features of AD.

A key distinction between research-grade and clinical-grade systems: research-grade prioritizes accuracy and complex feature mining; clinical-grade prioritizes speed (real-time inference ≤10 s), stability, ease of use, low cost, explainability, and regulatory compliance—core requirements for primary care and community screening. In clinical practice, AI-driven real-time MRI analysis systems have been applied beyond neurodegenerative diseases. For example, real-time MRI combined with deep learning-based segmentation has been used to assess dynamic wrist movement, providing quantitative measures of joint function, and clinical trials are still needed to confirm its value for the diagnosis of dynamic carpal instability (71). Similarly, real-time MRI with AI-based analysis has been employed to evaluate gastric motility, capturing physiological peristaltic parameters and differentiating between fasting and fed states (72). These applications illustrate the versatility of AI-powered real-time MRI analysis in diverse clinical contexts, enhancing diagnostic accuracy and workflow efficiency.

The robustness and generalizability of AI models in real-time MRI analysis depend on factors such as training dataset size, model architecture, and transfer learning strategies. Studies have shown that increasing the size of training cohorts improves the robustness of deep learning models for MRI attenuation correction in PET/MRI, with transfer learning enabling adaptation to different MRI sequences using limited additional data (73). Furthermore, weakly supervised deep learning frameworks with attention mechanisms have demonstrated excellent performance in scenarios with limited annotated data, representing a potential approach for clinical computer-aided diagnosis of AD (74). These methodological advances support the deployment of AI-driven real-time MRI analysis systems in routine clinical settings.

In summary, AI-driven real-time MRI image analysis and clinical auxiliary systems combine advanced deep learning techniques with high-resolution dynamic imaging to enable rapid, accurate, and automated diagnosis across multiple medical domains. These systems improve clinical workflows by reducing manual workload, enhancing diagnostic precision, and providing quantitative biomarkers for disease monitoring and treatment planning. Continued development and validation of such AI-powered frameworks are essential to realize their full potential in personalized medicine and real-time clinical decision support.

## 7 Conclusion

In conclusion, the integration of AI with MRI has profoundly advanced AD research and clinical practice, particularly in early detection and longitudinal progression monitoring. AI-driven methodologies, especially deep learning and machine learning, have automated complex MRI processing and subtle feature extraction, significantly improving diagnostic accuracy, workflow efficiency, and clinical scalability while reducing inter-observer variability.

Multimodal data fusion and large-scale multi-center databases (e.g., ADNI) have become pivotal for enhancing model generalizability and real-world applicability. By integrating structural, functional, and diffusion MRI with clinical and molecular biomarkers, AI models capture the multifaceted pathophysiology of AD more comprehensively than unimodal approaches, supporting robust algorithm training and accelerated clinical translation.

Nevertheless, critical challenges persist: cross-scanner/data heterogeneity, limited model interpretability, demographic bias (Western-dominated datasets), and unresolved ethical/privacy/regulatory concerns hinder widespread adoption. Addressing these requires standardized imaging protocols, rigorous external validation, and close multidisciplinary collaboration among clinicians, data scientists, and regulators. The 2026–2030 phased research roadmap illustrated in Figure 5 is not arbitrary speculation; each milestone is synthesized and inferred from published forward-looking reviews, multi-center AD-neuroimaging AI trend analyses and existing practical bottlenecks in this field (43, 55, 63). The staged timeline fully considers current constraints including ethnic dataset insufficiency, immature lightweight explainable-AI tools, scarcity of real-world clinical trial evidence and incomplete medical-AI regulatory systems.

**Figure 5:**
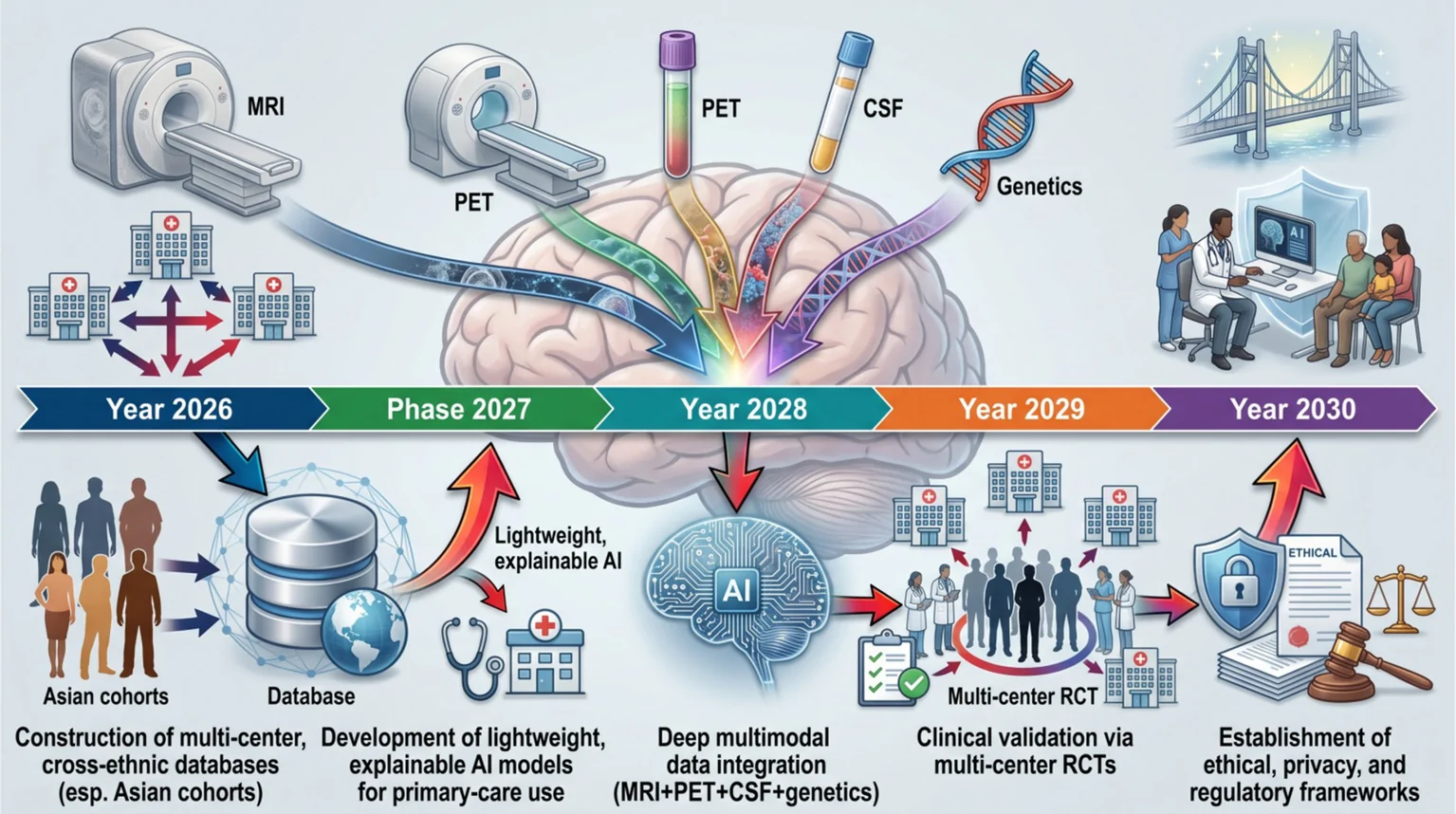
Evidence-based five-year research roadmap for AI-MRI research in Alzheimer’s disease (2026–2030). Each phased milestone is grounded on published trend analyses and bottleneck investigations of AD neuroimaging-AI (43, 55, 63). The timeline outlines five core priorities: (1) construction of multi-center, cross-ethnic databases (especially Asian cohorts); (2) development of lightweight, explainable AI models for primary-care deployment; (3) deep multimodal data integration (MRI + PET + CSF + genetics); (4) real-world clinical validation via multi-center RCTs; (5) establishment of supporting ethical, privacy, and regulatory frameworks. This roadmap serves as a prospective outlook to promote translation of AI-MRI technology from research into routine clinical practice, rather than deterministic prediction.

Over the next 3–5 years, several concrete research priorities and corresponding unresolved technical challenges will drive progress in AI-MRI for AD: (1) Construct multi-ethnic multi-center datasets, especially expanding Asian-population cohorts; unresolved challenge: harmonizing heterogeneous multi-scanner imaging data while preserving disease-related biological signals. (2) Develop lightweight, clinically-adaptable explainable-AI models; open challenges: standardizing XAI evaluation metrics and reducing computational overhead for primary-care deployment. (3) Advance multimodal fusion integrating MRI, multi-omics, clinical and textual data; unresolved problem: effective cross-modal alignment for heterogeneous high-dimensional data. (4) Perform prospective, multi-center real-world clinical validation rather than relying only on retrospective public-dataset performance; practical obstacle: high cost of prospective cohort recruitment and data curation. (5) Promote methodological research on federated learning, continual learning and medical foundation/vision-language models; key bottlenecks include domain adaptation and computational burden. (6) Establish complete ethical, privacy-protection and regulatory frameworks for dementia-oriented medical AI.

In summary, despite remaining hurdles, AI-MRI represents a transformative leap in AD care. It paves the way for earlier diagnosis, precise prognosis, and tailored interventions, ultimately improving quality of life for AD patients.
